# Long-Term Oncologic Outcomes After Laparoscopic and Robotic Tumor Enucleation for Renal Cell Carcinoma

**DOI:** 10.3389/fonc.2020.595457

**Published:** 2021-01-14

**Authors:** Wen Dong, Xiong Chen, Ming Huang, Xu Chen, Ming Gao, Dehua Ou, Kaiwen Li, Chenyang Wang, Shaoxu Wu, Hao Liu, Weibin Xie, Wenlian Xie, Steven C. Campbell, Tianxin Lin, Jian Huang

**Affiliations:** ^1^ Guangdong Provincial Key Laboratory of Malignant Tumor Epigenetics and Gene Regulation, Department of Urology, Sun Yat-sen Memorial Hospital, Sun Yat-sen University, Guangzhou, China; ^2^ Department of Radiology, Sun Yat-sen Memorial Hospital, Sun Yat-sen University, Guangzhou, China; ^3^ Department of Urology, Glickman Urological and Kidney Institute, Cleveland Clinic, Cleveland, OH, United States

**Keywords:** renal cell carcinoma, nephron sparing surgery, tumor enucleation, survival, follow-up

## Abstract

**Objectives:**

Tumor enucleation (TE) optimizes parenchymal preservation with promising short-term oncologic outcomes compared with standard partial nephrectomy (SPN). However, researches/literatures about long-term oncologic outcomes for TE after minimally invasive surgery are scarce. We aim to analyze long-term oncologic outcomes after laparoscopic and robotic tumor enucleation for renal cell carcinoma (RCC).

**Patients and Methods:**

We retrospectively analyzed 146 patients who underwent TE with either laparoscopic or robotic approach for localized RCC in our center. Local recurrence, cancer specific survival (CSS), recurrence free survival (RFS), and overall survival (OS) were the main outcomes. Survival curves were generated using a Kaplan-Meier method. Perioperative outcomes and pathological outcomes were also analyzed.

**Results:**

Overall, 98 male and 48 female patients were eligible for the study. The median tumor size was 3.4 cm with a median R.E.N.A.L. score of seven. Warm ischemia was used in 143 patients with a median ischemia time of 20 min and three patients had zero ischemia. Five patients (3.4%) had major complications (> Clavien IIIa) and only two were related to urinary system. The median global glomerular filtration rate (GFR) preserved after surgery was 93%. Pseudocapsule invasion was reported in 50 tumors (34%) and positive surgical margins were found in 3/146 (2.1%) tumors. At a median follow-up of 66 months, local recurrence happened in two patients (1.4%), and systemic recurrence happened in six patients (4.2%). The 5-year CSS, RFS, OS were 95.7, 89.6, and 91.9%, and the 10-year CSS, RFS, OS were 93.8, 89.6, and 90.0%, respectively.

**Conclusion:**

This study indicates that tumor enucleation with laparoscopic or robotic approach in experienced hands for the treatment of RCC appears oncologically safe with a median follow-up of more than 5 years. Prospective studies with more patients and longer follow-up will be required to further evaluate oncologic safety after TE.

## Introduction

Renal cell carcinoma (RCC) represents approximately 3% of all cancers, with an annual growth rate of 2% in incidence both worldwide and in Europe. As EAU and AUA guidelines recommended, surgery is the only curative treatment for localized RCC (1, 2). Surgery approaches include radical nephrectomy (RN) and nephron sparing surgery (NSS). Many studies have supported comparable cancer specific survival (CSS) for NSS *versus* RN (1–4). In order to preserve more renal units and general kidney function, NSS is recommended for RCCs with tumor <7 cm (1, 2). Approaches for NSS include standard partial nephrectomy (SPN) as well as tumor enucleation (TE). TE preserves more renal parenchyma by sharp combined with blunt dissection of the renal tumor along the plane between the pseudocapsule and the healthy renal tissue (5, 6). SPN excises about 0.5 to 1 cm healthy renal parenchyma (safety margin) around the tumor (7, 8). Many studies suggested that TE might be a better surgical technique for its preservation of more parenchyma without decreasing CSS and the anatomic landmarks it supplied during surgeries (9, 10). As a result, a growing number of clinical centers prefer to use TE but the trade-off between the advantages of preserving renal parenchyma and the oncologic outcomes remains debatable (6, 11, 12). Some retrospective studies reported the short-term oncologic outcomes of TE were comparable with SPN and RN (10, 11, 13). However, researches/literatures about long-term oncologic outcomes for TE after minimally invasive surgery are scarce. In this study we aim to analyze long-term oncologic outcomes after laparoscopic and robotic TE for RCC in a tertiary medical center, which may offer a good supporting evidence for using TE in localized RCC patients.

## Patients and Methods

### Patients

From 2008 to 2017, data of pathologically diagnosed RCC patients who received either laparoscopic or robotic TE at Sun Yat-sen Memorial Hospital were collected. All Surgeries were performed by two skilled urologists who have passed the learning curve for both laparoscopic and robotic partial nephrectomy. Study was approved by the local ethics committee and informed consent was obtained from each patient.

### Surgical Technique

For laparoscopic TE, as we described before, patients were placed in the flank position with three to four laparoscopic ports placed in standard retroperitoneal fashion (10). After removing extraperitoneal fat, incising the Gerota fascia and resecting the perirenal fat, the tumor and surrounding parenchyma were exposed. Then, the main renal artery was isolated and clamped by bulldog. The resection started from approximately 2 mm away from the tumor margin. When the pseudocapsule was identified, the surgeon took the pseudocapsule as anatomical landmark to enucleate the tumor by blunt together with sharp dissection from the surface to the bottom and then renal renorrhaphy was performed by using running sutures with one layer or two layers. According to the SIB scoring system for standardized reporting of NSS resection techniques, almost all of the tumors had a SIB score of less than 3, which means the tumors were resected mostly by pure enucleation or hybrid enucleation (14). For robotic TE, either transperitoneal approach or retroperitoneal approach was applied based on the surgeon’s preference. For all patients, a four-port technique was used, including a 12 mm trocar for camera, two 8 mm trocars for robotic arms, and a 12 mm trocar for the assistant. Robotic TE was performed similar as laparoscopic TE.

### Pathological Assessment

Tumors were classified according to the 2016 World Health Organization histologic classification system (15). The tumor stage and grade were determined according to the 2010 TNM system and the Fuhrman grading system (16). The margin status and pseudocapsule status were also evaluated.

### Outcome Parameters

Variables of patient characteristics were collected including age, sex, body mass index, Charlson comorbidity index, tumor size, and R.E.N.A.L. score. The perioperative data included surgical approaches, operative time, ischemia types, warm ischemia time, estimated blood loss, and major complications according to Clavien system. Serum creatinine was also assessed before and after operation and eGFR estimated by the MDRD2 equation for Chinese was calculated for analyzing renal function changes (17, 18).

All patients were regularly followed up in Sun Yat-sen Memorial Hospital. Chest X-ray or computed tomography (CT) scan was performed every year in the first 5 years. Abdominal CT scan was performed every 6 months in the first year and then once per year in the next 4 years. After 5 years of follow-up, CT scan was used every 2 years. Bone scan, brain CT, or magnetic resonance imaging (MRI) might be used in the presence of specific clinical or laboratory signs and symptoms. Oncological outcomes, such as local recurrence, CSS, and OS were also assessed.

### Statistical Analysis

Categorical variables were expressed as numbers with percentages and compared using chi-squared test or Fisher exact test. Continuous variables were presented as medians with interquartile ranges (IQRs) and compared using a Mann-Whitney U-test. Survival curves were generated using a Kaplan-Meier method. All values reported were two-sided with statistical significance defined as P < 0.05. All statistical analyses were performed using SPSS version 20.0 (SPSS Inc., Chicago, IL, USA).

## Results

Patient characteristics were summarized in **Table 1**. Totally, 146 patients with a median preoperative global GFR of 95 ml/min/1.73m^2^ (IQR: 84–106 ml/min/1.73 m^2^) were involved. The median age of these patients was 53 y (IQR: 42 y–62 y) and 98 (67.1%) patients were male. Median body mass index was 24.5 kg/m^2^ (IQR: 22.0–26.7 kg/m^2^) and median Charlson comorbidity index was 0 (IQR: 0–1). Median clinical tumor size was 3.4 cm (IQR: 2.6–4.6 cm) with a median R.E.N.A.L. score of 7 (IQR: 6–9). Among them, 56 (38.4%) were low tumor complexity, 55 (37.7%) were intermediate, and 35 (23.9%) were high. Three patients had solitary kidney.

**Table 1:** Patient characteristics.

Number of patients	146
Age (years) (median, IQR)	53 (42–62)
Male (%)	98 (67.1)
BMI (kg/m^2^) (median, IQR)	24.5 (22.0–26.7)
CCI (median, IQR)	0 (0–1)
Clinical tumor size (cm) (median, IQR)	3.4 (2.6–4.6)
R.E.N.A.L. score (median, IQR)	7 (6–9)
Tumor complexity (%)	
Low (R.E.N.A.L 4-6)	56 (38.4)
Intermediate (R.E.N.A.L 7-9)	55 (37.7)
High (R.E.N.A.L 10-12)	35 (23.9)
Preoperative global GFR (mL/min/1.73m^2^) (median, IQR)	95 (84–106)
Solitary kidney (%)	3 (2.1)

BMI, body mass index; CCI, Charlson comorbidity index; GFR, glomerular filtration rate; IQR, interquartile range; R.E.N.A.L., (R)adius (tumor size as maximal diameter), (E)xophytic/endophytic properties of tumor, (N)earness of tumor deepest portion to collecting system or sinus, (A)nterior (a)/posterior (p)descriptor and (L)ocation relative to polar lines.

Perioperative, pathological, and functional outcomes were reported in **Table 2**. With a median operative time of 130 min (IQR: 100–180 min), laparoscopic TE was applied to 115 patients while robotic TE was used for 31 patients. Three patients had zero ischemia while the others had warm ischemia with a median ischemia time of 20 min (IQR: 15–28 min). Median estimated blood loss was 50 ml (IQR: 20–100 ml) and transfusion was required in two patients. According to Clavien system, major complications (≥ Clavien IIIa) happened to five patients and only two were related to urinary system. At pathological assessment, 119 patients were confirmed clear cell carcinoma, 15 were papillary, nine were chromophobe, and three were other types. Fuhrman grade in clear cell carcinoma resulted ≤II in 85 (71.4%) and ≥III in 34 (28.6%). As for pathological tumor stage, 94 (64.4%) patients were pT1a, 46 (31.5%) were pT1b, 4 (2.7%) were pT2, and 2 (1.4%) were pT3a. Pseudocapsule can be seen in each tumor. Pseudocapsule invasion was found in 50 patients and positive margins occurred in three patients. After surgery, median new baseline global GFR was 88 ml/min/1.73 m^2^ (IQR: 73–100 ml/min/1.73 m^2^) which meant median global GFR preserved was 93% (IQR: 86–98%).

**Table 2:** Perioperative, pathological, and functional outcomes.

Number of patients	146
Surgical approach (%)	
Laparoscopic	115 (78.8)
Robotic	31 (21.2)
Transfer to open surgery	1 (0.7)
Operative time (minutes) (median, IQR)	130 (100–180)
Warm ischemia time (minutes) (median, IQR)	20 (15–28)
Ischemia type (%)	
Zero	3 (2.1)
Warm	143 (97.9)
EBL (ml) (median, IQR)	50 (20–100)
Transfusion (%)	2 (1.4%)
Major complications ( ≥ Clavien IIIa)	5 (3.4%)
Histology	
Clear cell	119 (81.5)
Papillary	15 (10.3)
Chromophobe	9 (6.1)
Other	3 (2.1)
Fuhrman grade (%) (for ccRCC)	
≤II	85 (71.4)
≥III	34 (28.6)
Pathological tumor stage (%)	
pT1a	94 (64.4)
pT1b	46 (31.5)
pT2	4 (2.7)
pT3a	2 (1.4)
Pseudocapsule invasion (%)	50 (34.2)
Positive margins (%)	3 (2.1)
New baseline global GFR (ml/min/1.73 m^2^) (median, IQR)	88 (73–100)
GFR preserved (%) (median, IQR)	93 (86–98)

ccRCC, clear cell renal cell carcinoma; EBL, estimated blood loss; IQR, interquartile range.

**Figure 1:**
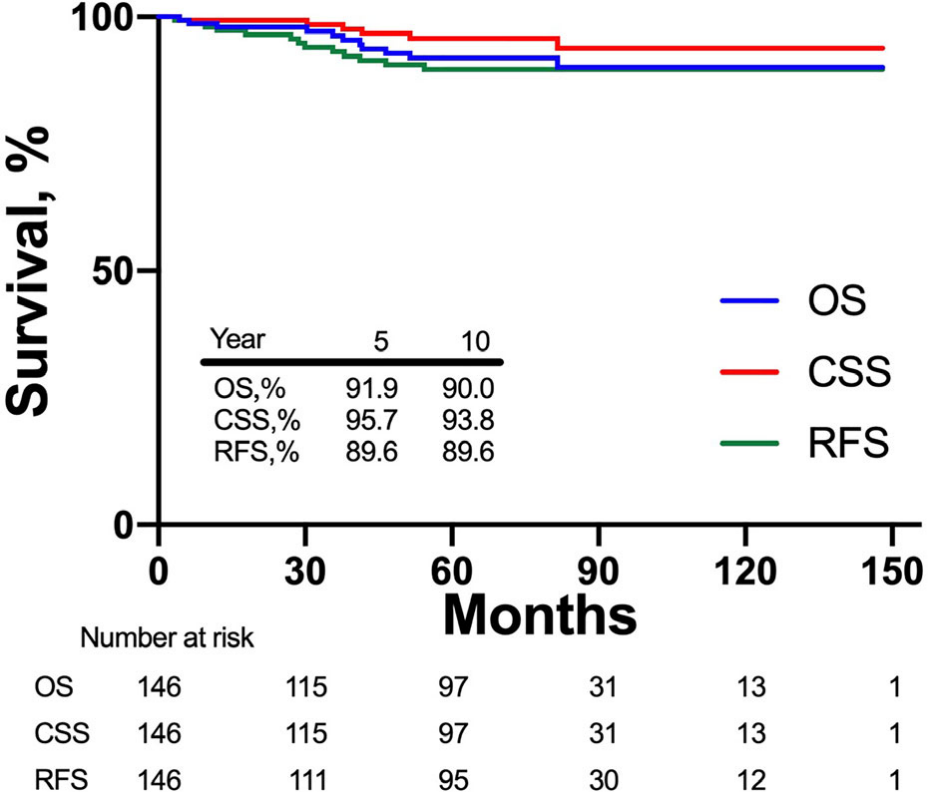
Kaplan-Meier curves depicting overall survival (blue), cancer specific survival (red) and recurrence free survival (green) in 146 patients treated with laparoscopic and robotic enucleation for renal cell carcinoma.

At a median follow-up of 66 months, local recurrence happened to two patients and systemic recurrence happened to six patients (**Table 3**). Among patients with local recurrence, one happened at tumor bed while the other one happened in elsewhere in the ipsilateral kidney. As for systemic recurrence, two were detected in bone, two in lung, one in peritoneal, and one in contralateral adrenal gland. Besides, five patients died due to non-cancer causes. **Figure 1** shows CSS, RFS, and OS in patients after laparoscopic and robotic tumor enucleation for localized RCC according to time after surgery. The 5-year CSS, RFS, OS were 95.7, 89.6, and 91.9%, and the 10-year CSS, RFS, OS were 93.8, 89.6, and 90.0%, respectively.

**Table 3:** Oncologic outcomes.

Number of patients	146
Median follow-up (months)	66
Local recurrence (%)	2 (1.4)
Tumor bed	1 (0.7)
Elsewhere in the ipsilateral kidney	1 (0.7)
Systemic recurrence (%)	6 (4.2)
Bone	2 (1.4)
Lung	2 (1.4)
Peritoneal	1 (0.7)
Contralateral adrenal gland	1 (0.7)
RCC related mortality	6 (4.2)
Other cause mortality	5 (3.4)

RCC, renal cell carcinoma.

## Discussion

TE has gained growing acceptance among urologists for excellent perioperative results and short-term oncologic outcomes (5, 6, 19). Studies also imply that TE can preserve more normal renal parenchyma which is associated with lower risk of post-operative kidney diseases (20, 21). It is widely accepted that better renal function is beneficial for cardiovascular system and associated with longer overall survival (1–3).

In this study, we had 146 patients performed laparoscopic or robotic TE, and to our knowledge our study is the longest median follow-up to evaluate long-term oncologic outcomes of TE by minimally invasive surgeries. The 5-year and 10-year OS were 91.9 and 90.0% respectively. The 5-year CSS in our study was 95.7% which was comparable to some other studies (13, 22, 23). The 10-year CSS in our study was also up to 93.8% which means we seldom found patients died of cancer more than 5 years after TE. At a median follow-up of 66 months, two patients (1.4%) had local recurrence and only one of them had recurrence at the tumor bed, and six patients (4.1%) had systemic recurrences.

TE preserves more parenchyma, which may contribute to a better renal function after surgery (20, 21). In our study, median global GFR preserved 1 year after surgery was up to 93% (IQR: 86–98%) while it was about 89% in SPN reported in other studies (24, 25). However, a closer distance from the resection plane to tumor makes some urologists uncomfortable because they are worried about the positive margins and local recurrences that might happen in TE patients. Although level 1 evidence is lacking to prove that positive margins in TE patients are comparable to SPN patients, many retrospective studies, review articles, and prospective non-randomized studies imply that TE is as safe as SPN with very few positive margins and local recurrences for localized renal cell carcinomas (10, 26–29).

In the era of open surgery, Carini et al. reported the long-term follow-up of TE for T1a and tumor size between 4 and 7 cm renal cell carcinoma separately (22, 23). At a median follow-up of 61 months and mean follow-up of 76 months for T1a tumors, the 5-year and 10-year cumulative survival rates were 89.5 and 81.4%, and the 5-year and 10-year CSS were 96.7 and 94.7%, respectively. Overall, 6.4% patients had disease progression, three of whom had local recurrences alone (1.5%) elsewhere in the kidney; none had local recurrence at the enucleation bed. At a median follow-up of 51 months and mean follow-up of 74 months for tumor size between 4 and 7 cm, 5-year and 8-year CSS were 85.1 and 81.6%, respectively. Overall 10 patients experienced progressive disease (14.9%), of whom three had local recurrence (4.5%) alone or local recurrence associated with distant metastases. Minervini recently reported a group of 127 robotic TE patients and the positive surgical margins were found in three patients (2.4%). After a median follow-up of 61 months, no recurrence cases at the enucleation site were recorded, and three cases (2.4%) had renal recurrence elsewhere in the ipsilateral kidney. They also found a distinct peritumoral pseudocapsule was presented in 121/127 (95%) tumors, among which partial and complete pseudocapsule invasion was reported in 49/121 (40.5%) and 24/121 (19.8%) cases, respectively (26).

While in our study the rate of positive surgical margin was merely 2.1%, and as we described above, local recurrence was also rarely seen in TE. We could find pseudocapsule in all cases and pseudocapsule invasion in 50 (34%) cases, which was similar to what we had reported several years ago (10). We seldom found complete pseudocapsule invasion in our patients mostly because the T3a patients in our study only accounted for 1.4%. Based on our experience, positive surgical margin and local recurrence are not related to TE technique but the surgeon’s experience. TE is applied to patients after carefully taking into account of the tumor characteristics including growth pattern and interface with normal parenchyma as long as negative surgical margins are prioritized in our center. For beginners, we highly recommend starting with small exophytic tumors surrounded by a distinct pseudocapsule from the images.

Our data also demonstrated that high complexity tumors accounted for about 24% in this study which was higher than data from other studies (19, 26). Our study may have implications for surgical technique as it suggests TE can also be safely used for renal hilar tumors. With the application of robotic surgery and increasing experience in TE, we find TE is the best option to avoid the kidney being completely removed or a large amount of healthy renal parenchyma being devascularized for tumors located in the renal hilum or adjacent to large vessels. Tumor diameter and endophytic status were significantly associated with complete pseudocapsule invasion according to published research (26). In order to avoid positive surgical margins in these complex cases, especially large and endophytic tumors located in the renal hilum, we always clamp the main renal artery and sometimes clamp the renal vein to keep a bloodless field. We also use ultrasound when the boundary of the tumor is difficult to find.

Despite its strengths, our study has some limitations. First, its retrospective and single center design, and the tertiary care patient population could impact generalizability. Second, although laparoscopic and robotic tumor enucleation are both belong to minimally invasive surgery, most studies indicate robotic surgery is superior than laparoscopic surgery in perioperative outcomes (30). It is unclear whether this potential superiority could affect the long-term oncologic outcomes although the surgeons in this study are very good at both approaches. inally, given the natural history of renal cell carcinoma, the relatively small sample size especially only 31 procedures performed with robotic surgery and the median follow-up of 66 months in our study might still be not enough to assess potential delayed recurrences after laparoscopic and robotic TE.

In conclusions, our study indicates that TE for RCC patients either by laparoscopic or robotic approach in experienced hands are oncologically safe with a median follow-up of more than 5 years. TE can preserve more parenchyma and achieve negative surgical margins even in complex cases due to the existence of pseudocapsule in the vast majority of tumors. Prospective studies with more patients and longer follow-up will be required to further evaluate oncologic safety after TE.

## Data Availability Statement

The raw data supporting the conclusions of this article will be made available by the authors, without undue reservation.

## Ethics Statement

The studies involving human participants were reviewed and approved by Sun Yat-sen Memorial Hospital ethics committee. The patients/participants provided their written informed consent to participate in this study.

## Author Contributions

WD and XiC prepared the manuscript, which was reviewed by all authors. All authors contributed to the article and approved the submitted version.

## Funding

The study was supported by the National Natural Science Foundation of China (Grant No. 81972383, U1301221, 81702523); Natural Science Foundation of Guangdong (2019A1515010188, 2020A1515010888).

## Conflict of Interest

The authors declare that the research was conducted in the absence of any commercial or financial relationships that could be construed as a potential conflict of interest.

## Abbreviations

CSS, cancer specific survival; CT, computed tomography; EBL, estimated blood loss; GFR, glomerular filtration rate; IQR, interquartile range; MRI, magnetic resonance imaging; NSS, nephron sparing surgery; OS, overall survival; RCC, renal cell carcinoma; R.E.N.A.L., (r)adius (tumor size as maximal diameter), (e)xophytic/endophytic properties of tumor, (n)earness of tumor deepest portion to collecting system or sinus, (a)nterior (a)/posterior (p) descriptor, and (l)ocation relative to polar lines; RFS, recurrence free survival; RN, radical nephrectomy; SPN, standard partial nephrectomy; TE, tumor enucleation; WIT, warm ischemia time.
